# Association Between Embodiment of a Muscular Avatar and Muscle Performance in Virtual Reality for Chronic Low Back Pain: Exploratory Pre-Post Study

**DOI:** 10.2196/90389

**Published:** 2026-10-09

**Authors:** Yuichiro Nakaso, Yong-Hao Hu, Yukiko Shiro, Masayuki Inoue, Takafumi Kanaya, Takuto Nakamura, Takuji Narumi, Takahiro Ushida

**Affiliations:** 1 Department of Pain Medicine, Aichi Medical University, 1-1, Yazako karimata, Nagakute, Aichi, 480-1195, Japan, 81 561-62-5004; 2 Graduate School of Information Science and Technology, The University of Tokyo, Bunkyo, Tokyo, Japan; 3 Department of Physical Therapy, Faculty of Rehabilitation Sciences, Nagoya Gakuin University, Nagoya, Aichi, Japan; 4 Institute of Physical Fitness, Sports Medicine and Rehabilitation, School of Medicine, Aichi Medical University, Nagakute, Aichi, Japan; 5 Graduate School of Interdisciplinary Information Studies, The University of Tokyo, Bunkyo, Tokyo, Japan; 6 D3 Center, Osaka University, Suita, Osaka, Japan

**Keywords:** chronic low back pain, virtual reality, muscle strength, muscular avatar, embodiment, proteus effect, exercise, rehabilitation

## Abstract

**Background:**

Chronic low back pain (CLBP) is associated with impaired muscle performance, including reduced trunk strength, as well as disturbances in body perception. Virtual reality (VR) enables modulation of body representation, and the Proteus effect suggests that embodying a stronger avatar may influence perceived bodily capability and behavior. Although preliminary CLBP studies have reported short-term improvements in pain and beliefs, it remains unclear whether avatar-based embodiment can acutely modify muscle strength or relate to subjective embodiment.

**Objective:**

This study aimed to examine immediate changes in muscle strength and pain after a VR-based squat task with a progressively muscular avatar in individuals with CLBP and explore associations between strength changes and subjective embodiment.

**Methods:**

In this exploratory pre-post proof-of-concept study, 16 individuals with CLBP and 16 age- and sex-matched healthy controls performed a VR-based squat task with a progressively muscular avatar. Muscle strength (handgrip, trunk extension, and knee extension) was assessed before and after VR. Pain and fear were assessed in CLBP only. Subjective embodiment was assessed using the Virtual Embodiment Questionnaire (VEQ). Group × time effects were analyzed using repeated-measures ANOVA, and associations were analyzed using Spearman correlations with false discovery rate correction. All participants completed the VR-based task and assessments, and no missing data were observed in variables included in the analyses.

**Results:**

Significant time effects were observed for handgrip strength (*P*=.04), trunk extension strength (*P*<.001), and knee extension strength (*P*<.001), whereas no significant group × time interactions were observed for handgrip, trunk extension, or knee extension strength (*P*=.75, *P*=.19, and *P*=.28, respectively). Trunk extension strength significantly increased in healthy controls (mean difference 0.04 kgf/kg, 95% CI 0.03 to 0.05; Bonferroni-adjusted *P*<.001) but not in individuals with CLBP (mean difference 0.02 kgf/kg, 95% CI 0.001-0.05; Bonferroni-adjusted *P*=.08). Knee extension strength increased in both the CLBP group (mean difference 0.06 kgf/kg, 95% CI 0.02-0.11; Bonferroni-adjusted *P*=.02) and healthy controls (mean difference 0.09 kgf/kg, 95% CI 0.06-0.12; Bonferroni-adjusted *P*<.001). In the CLBP group, pain intensity decreased during VR compared with pre-VR levels (*P*=.02), whereas post-VR pain did not differ from pre-VR levels. Changes in trunk extension strength were positively correlated with VEQ change in the CLBP group (ρ=0.76, 95% CI 0.41-0.94; false discovery rate–adjusted *P*=.006).

**Conclusions:**

Trunk and knee extension strength showed time-related increases without significant group × time interactions, and trunk extension strength change was associated with VEQ change in the CLBP group. These findings suggest that avatar-based modulation of body perception may relate to individual variability in motor output. However, without a neutral-avatar control condition, the observed changes cannot be attributed specifically to the progressive muscular transformation of the avatar. Further research is needed to clarify the role of avatar-based embodiment in exercise-based rehabilitation.

## Introduction

### Problem

Low back pain (LBP) is one of the most prevalent musculoskeletal conditions and a leading cause of long-term disability worldwide [1,2]. Chronic low back pain (CLBP) is a persistent and clinically important form of this condition. Beyond persistent pain, individuals with CLBP commonly demonstrate motor impairments, including reduced trunk extensor strength, altered muscle activation, and increased trunk stiffness [3-5]. Exercise therapy is a core component of CLBP management and provides small-to-moderate improvements in pain and function [6]. However, some patients show limited responses to conventional exercise therapy, suggesting that neurophysiological and perceptual factors may need to be considered when addressing persistent symptoms.

### Review of Relevant Scholarship

A growing body of evidence indicates that pain can alter voluntary motor output. Hodges and Tucker [7] proposed that adaptation to pain involves redistribution of activity within and between muscles, changes in movement and stiffness, and alterations across multiple levels of the motor system, rather than simple changes in excitability. Consistent with this framework, individuals with CLBP show motor control changes, including altered muscle activity, trunk stiffness, and motor variability [8], as well as altered spinal protective motor responses [9]. These adaptations may limit efficient motor output and contribute to reduced muscle performance.

In addition to altered motor control, CLBP is associated with disturbances in body perception. Neuroimaging studies have reported structural and functional alterations in cortical regions involved in sensorimotor processing and body representation in individuals with CLBP [10,11]. Clinically, patients may experience impaired tactile acuity and distorted back-specific body image, suggesting less precise representation of the painful back within the nervous system [12]. A recent systematic review reported that disturbances in body awareness in individuals with LBP were associated with greater pain severity, pain catastrophizing, and disability [13]. These findings suggest that body perception, in addition to muscle performance, may represent a clinically relevant target for some individuals with CLBP.

Virtual reality (VR) offers a unique opportunity to target these interacting perceptual and motor processes by enabling immersive, real-time manipulation of one’s virtual body. A central concept in this literature is the Proteus effect, which provides a theoretical framework for understanding how avatar characteristics shape user attitudes, behaviors, and performance in virtual environments [14,15]. Recent experimental work in healthy adults has shown that embodying a more muscular avatar can reduce experimentally induced pain, suggesting that visually enhanced body representation may influence pain perception [16]. In individuals with CLBP, preliminary studies have reported that embodying a superhero-like avatar can influence pain-related outcomes, back-related perceptions, and perceived bodily competence [17], and that manipulating visual-proprioceptive feedback in VR can alter movement-evoked pain and pain-free range of motion [18]. Recent feasibility work has also suggested that immersive VR programs combining movement exercises, real-time feedback, and body illusions are acceptable and may improve pain-related and functional outcomes in chronic back pain [19]. However, it remains unclear whether embodiment of an avatar with enhanced physical characteristics can influence objective muscle performance, particularly voluntary muscle strength, in individuals with CLBP.

### Hypothesis, Aims, and Objectives

Accordingly, as an early-stage, proof-of-concept study grounded in body-representation theories, we aimed to investigate whether embodying a progressively muscular avatar during a VR-based exercise task could induce short-term changes in muscle strength and pain intensity in patients with CLBP. We further examined whether these changes were associated with subjective experiences of virtual embodiment. We hypothesized that embodying a progressively muscular avatar would acutely increase muscle strength without exacerbating pain intensity, and that the magnitude of strength changes would be associated with the subjective experience of virtual embodiment.

## Methods

### Study Design

This exploratory proof-of-concept study used a 2-group pre-post design involving individuals with CLBP and age- and sex-matched healthy controls. All participants underwent muscle strength and self-reported assessments immediately before and after a single VR-based squat task involving embodiment of a progressively muscular avatar. The study was designed to examine acute changes in muscle strength, pain intensity, and other pain-related outcomes and their associations with subjective virtual embodiment experiences. This study did not include a neutral-avatar control condition, placebo condition, or blinding procedure. Accordingly, the design was not intended to estimate the causal effect of progressively muscular avatar embodiment.

### Inclusion and Exclusion

The inclusion criteria for the CLBP group were as follows: (1) age ≥18 years, (2) LBP lasting for ≥3 months, and (3) an average LBP intensity of ≥3 on the 11-point Numerical Rating Scale (NRS) over the past week at baseline.

The inclusion criteria for the healthy control group were as follows: (1) age ≥18 years, (2) no current LBP requiring medical care, and (3) no history of CLBP lasting for ≥3 months.

The exclusion criteria for both groups were as follows: (1) musculoskeletal, neurological, visual, or vestibular impairments that made it difficult or unsafe to perform the VR-based squat task, including suspected or confirmed lumbar radiculopathy or neurological deficits; (2) severe cognitive or psychiatric conditions that hindered understanding of the study procedures or provision of informed consent; (3) conditions that could interfere with safe VR exposure, such as severe motion sickness, photosensitive epilepsy, or severe vestibular symptoms; or (4) any other condition judged by the principal investigator to pose a safety concern or prevent completion of the study protocol.

### Participant Characteristics

Participant characteristics, including age and sex for both groups and pain-related clinical variables for the CLBP group, are summarized in Table 1. Most participants had no identified specific cause of LBP and were considered to have nonspecific CLBP. A small number had degenerative spinal diagnoses, such as lumbar spondylosis, but none had lumbar radiculopathy, neurological deficits, or other specific spinal pathologies that clearly accounted for their LBP.

**Table 1.** Demographic and baseline characteristics. Values are presented as mean (SD), n (%), or median (IQR), as appropriate.

Variables	CLBP^a^ (n=16)	Healthy controls (n=16)	*P* value
Age (years), mean (SD)	56.9 (17.4)	56.5 (16.8)	.94
Sex, n (%)			>.99
Female	7 (43.8)	7 (43.8)	
Male	9 (56.2)	9 (56.2)	
BMI, n (%)			.52
<20 kg/m^2^	5 (31.2)	2 (12.5)	
20‐25 kg/m^2^	8 (50)	9 (56.2)	
>25 kg/m^2^	3 (18.8)	5 (31.3)	
Duration of low back pain (months), median (IQR)	120 (66.8‐238.5)	—^b^	—
Pain intensity average last week (NRS)^c^, median (IQR)	4 (3‐6)	—	—
RDQ^d^, median (IQR)	5 (3.8‐7.3)	—	—
FreBAQ^e^, median (IQR)	6.5 (4.8‐10.3)	—	—

a CLBP: chronic low back pain.

b N/A: not applicable.

c NRS: Numeric Rating Scale.

d RDQ: Roland-Morris Disability Questionnaire.

e FreBAQ: Fremantle Back Awareness Questionnaire.

### Sampling Procedures

Participants with CLBP were recruited using convenience sampling from the outpatient clinic of the Pain Center at Aichi Medical University Hospital, whereas healthy controls were recruited through flyer advertisements posted within the facility. All assessments, including muscle strength measurements, self-reported questionnaires, and the VR-based squat task, were conducted at Aichi Medical University. After enrollment of the CLBP group, healthy controls were selected to match the CLBP group by age (±5 y) and sex. All enrolled participants completed the VR-based squat task and all assessments; therefore, all were included in the final analysis.

### Sample Size, Power, and Precision

Sample size considerations were based on the main muscle strength outcomes. An a priori power analysis was performed using G*Power (version 3.1; Heinrich Heine University) for a 2-way repeated-measures ANOVA (group × time) with 2 groups and 2 measurements (pre and post). Assuming a medium-sized within–between interaction effect (Cohen *f*=0.25), a 2-sided *α*=.05, and a correlation of *r*=0.50 between repeated measures (*ε*=1.0), a total sample size of 34 participants was required to achieve 80% power. Based on the proof-of-concept nature of this study, 32 participants (16 per group) were enrolled. Pain outcomes assessed at three time points (pre, during, and post) were exploratorily analyzed within the CLBP group and were not used for sample size determination. Therefore, this study had limited statistical power for detecting group × time interaction effects and exploratory correlations, and these findings were interpreted with consideration of effect sizes and 95% CIs.

### VR-Based Squat Task

The participants performed repeated squat exercises while observing a virtual avatar that moved in real time in synchrony with their own body movements. The VR environment was implemented using the Unity engine and displayed on a large monitor connected to a motion-tracking sensor system. The avatar was sex-matched to each participant to enhance identification and embodiment.

Before the task, the participants received standardized instructions: “You will first observe the avatar displayed on the screen. Subsequently, while continuing to observe the avatar, you will perform squats for 30 seconds. After the squatting task, please take a moment to experience the changes in the avatar.” Each squat session consisted of three consecutive phases: a 30-second observation phase, a 30-second squat exercise phase performed while continuing to observe the avatar, and a subsequent 30-second postexercise observation phase, allowing the participants to view the updated avatar appearance. During the squat phase, participants performed squats at a self-selected comfortable pace for 30 seconds. Because the task was time-based rather than repetition-based, the number of squats was not predetermined; however, the number completed during the task was recorded. During the task, the avatar’s muscularity gradually increased across the squat cycles, resulting in a dynamic visual transformation of its appearance. Participants were not informed in advance about these changes or their purpose to minimize demand characteristics and expectancy effects (Figure 1). After completing the VR intervention, participants were asked whether they had noticed a change in the avatar’s muscularity.

**Figure 1.**
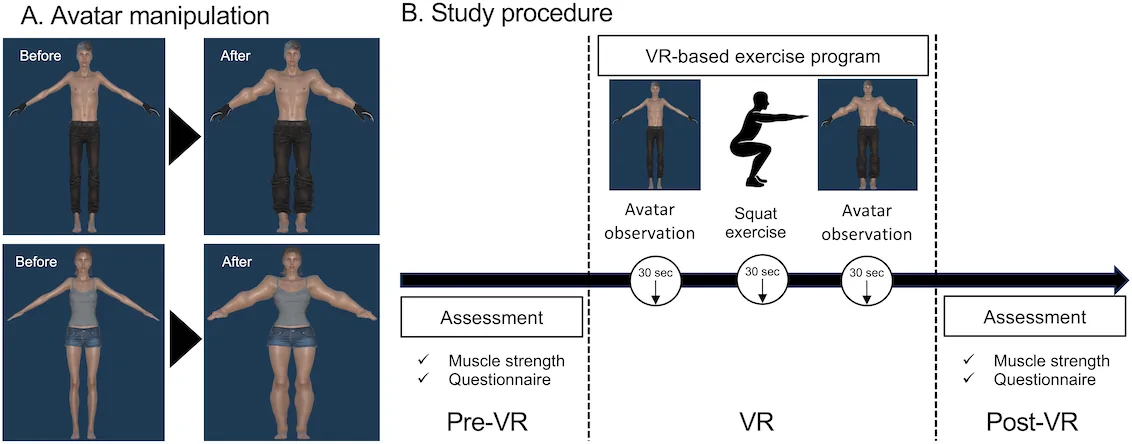
Overview of the avatar-based virtual reality intervention. (A) The participants embodied a self-avatar whose muscular appearance was progressively enhanced during the task, consistent with a Proteus effect–based manipulation. Both male and female avatars were used. (B) Single-session study procedure. After the baseline assessments of muscle strength and questionnaires, the participants completed a VR-based exercise program consisting of an initial avatar observation phase, a 30-second squat exercise performed while observing the avatar, and a postexercise avatar observation phase. Muscle strength and questionnaire measures were reassessed immediately after virtual reality exposure. VR: virtual reality.

### Measures and Covariates

#### Muscle Strength Measurement

Muscle strength was evaluated by measuring the maximal isometric strength for handgrip, trunk extension, and knee extension. Knee extension strength and trunk extension strength were included because the VR-based task primarily involved squat-related lower-extremity and trunk movements. Handgrip strength was included as a reference outcome because it represented a nontask-specific upper-extremity measure. All measurements were performed on the right side only to ensure procedural consistency across participants and are expressed in kilogram-force (kgf), consistent with the output of the dynamometer. Handedness was not formally assessed in this study.

#### Handgrip Strength

Handgrip strength was measured using a digital hand dynamometer (T.K.K. 5401; Takei Scientific Instruments). The participants stood upright in a relaxed posture with their arms at their sides to avoid contact with the trunk. Two maximal voluntary contractions were performed, each lasting approximately 3 seconds. Verbal encouragement was provided during each trial, and a 60-second rest interval was allowed between trials. The higher of the 2 values was used for the analysis.

#### Trunk Extension Strength

Isometric trunk extension strength was measured in the seated position using a belt-stabilized handheld dynamometer (Mobie; Sakai Medical), following the validated method described by Sawa et al [20]. The participants sat upright with their pelvis fixed with a belt, knees flexed at 90°, and arms crossed over the chest. A towel was placed on the sacrum to maintain a neutral pelvic tilt, and another towel supported the knees to reduce lower limb involvement. The participants performed 2 maximal isometric trunk extensions, each for 5 seconds, with a 60-second rest in between. The higher value was recorded.

#### Knee Extension Strength

Isometric knee extension strength was assessed using a belt-stabilized handheld dynamometer (Mobie; Sakai Medical), following the validated method described by Ushiyama et al [21]. The participants sat with their hips and knees flexed to 90° and stabilized with straps across the pelvis and thighs. The dynamometer was fixed to the distal part of the right lower leg (approximately 3 fingers above the malleolus) and anchored to a fixed pole using a nonelastic strap. The participants performed two 5-second maximal voluntary contractions of the right knee, with 60 seconds of rest between trials. The higher of the 2 values was used for the analysis.

### Pain and Disability Measures

To determine eligibility, the participants reported their average LBP intensity over the past week at baseline. In addition, the pain intensity at each time point was assessed immediately before, during, and after the VR-based squat task to capture task-related changes in pain. Pain severity was assessed using the NRS, where 0 indicated no pain, and 10 indicated the worst imaginable pain. The NRS is a widely used tool with established reliability and validity for assessing pain intensity [22].

The Roland-Morris Disability Questionnaire (RDQ) was used to assess disability related to LBP, with higher total scores indicating greater disability. The RDQ has been validated in both the original [23] and Japanese versions [24]. The RDQ was administered only in the CLBP group at baseline.

### Psychological Measures

Task-specific fear of movement during the squat task was assessed using an 11-point NRS, where 0 indicated no fear and 10 the highest possible degree of fear [25]. Task-specific measures of pain-related fear have been recommended over general measures when examining the relationship with movement behavior in individuals with CLBP [26]. Fear ratings were obtained in the CLBP group immediately before, during, and after the VR-based squat task.

### Body Perception and Embodiment Measures

The Fremantle Back Awareness Questionnaire (FreBAQ) was used to evaluate disturbances in body perception related to the back, with higher scores indicating more severe perceptual disturbances. The FreBAQ has demonstrated good reliability and validity in its original [27] and Japanese versions [28]. The FreBAQ was administered only in the CLBP group at baseline. Virtual embodiment was assessed using a Japanese-translated version of the Virtual Embodiment Questionnaire (VEQ) [29], based on the original VEQ [30]. Although the psychometric validation of this translated version has not yet been reported, we analyzed the 3 subscales (ownership, agency, and change) separately, in accordance with the original study. The VEQ was administered after completion of the VR-based exercise session in both groups.

### Data Collection

All participants underwent muscle strength testing before and immediately after the VR-based squat task. Participants in the CLBP group additionally completed self-reported measures of LBP intensity, disability, back-related body perception, and task-specific fear. Pain intensity and fear of squat motion in the CLBP group were assessed immediately before, during, and after the VR-based squat task. After completion of the VR-based squat task, participants in both groups completed the VEQ.

### Quality of Measurements

Standardized procedures were used for all measurements. Muscle strength assessments were performed using predefined positions, standardized contraction durations, and fixed rest intervals. Validated Japanese versions were used for questionnaire measures when available.

### Instrumentation

The VR environment was implemented using the Unity engine and displayed on a large monitor connected to a motion-tracking sensor system. Muscle strength was assessed using a digital hand dynamometer (T.K.K. 5401; Takei Scientific Instruments) and a belt-stabilized handheld dynamometer (Mobie; Sakai Medical). Detailed measurement procedures are described in the corresponding outcome sections above.

### Masking

Participants and researchers were not blinded to group status or the general study procedures. However, participants were not informed in advance that the avatar’s muscularity would progressively change or of the purpose of this manipulation, in order to minimize expectancy effects and demand characteristics.

### Data Diagnostics

Distributional assumptions were assessed using the Shapiro-Wilk test. No post-data-collection exclusions were applied, and no missing data required imputation. No data transformations were performed. No data were excluded as statistical outliers.

### Analytic Strategy

All analyses were performed using R (version 4.3.2; R Foundation for Statistical Computing). Continuous variables are summarized as mean (SD) or median (IQR), depending on the distribution.

To examine immediate changes in muscle strength before and after the VR-based squat task, analyses were primarily performed using body weight-normalized strength values (kgf/kg). Two-way repeated-measures ANOVAs (group × time) were performed for each outcome (handgrip, trunk extension, and knee extension), with group (CLBP vs healthy controls) as the between-subjects factor and time (pre vs post) as the within-subjects factor (type III sums of squares). Effect sizes for ANOVA are reported as partial η². The primary inferential focus for the muscle strength outcomes was the group × time interaction. In the absence of a significant group × time interaction, the main effects of time and within-group pre-post comparisons were interpreted as exploratory time-related changes rather than evidence of a differential task-related effect in the CLBP group. Planned within-group pre-post comparisons were performed using paired 2-tailed *t* tests, with Bonferroni adjustment applied across the 2 within-group comparisons for each outcome.

In the CLBP group, changes in pain intensity and task-specific fear assessed at multiple time points (pre, during, and post) were examined using the Friedman test, followed by the Bonferroni-adjusted Wilcoxon signed-rank test for pairwise comparisons. Kendall *W* was reported as the effect size for the Friedman test.

For each group, mean differences with 95% CIs were calculated. Within-group effect sizes were computed as Cohen *d*_av (standardized mean change using the average of the pre- and post-VR SDs). By convention, d_av values of approximately 0.2, 0.5, and 0.8 were interpreted as small, moderate, and large effects, respectively.

Associations between changes in muscle strength and VEQ subscales (ownership, agency, and change) were examined using the Spearman rank correlation coefficient (ρ). CIs were estimated using bootstrap resampling (5000 iterations). To account for multiple testing, *P* values were adjusted using the Benjamini-Hochberg false discovery rate (FDR) procedure within each group (9 correlations per group). All statistical tests were 2-tailed, with significance set at *α*=0.05. Given the exploratory proof-of-concept nature of this study and the multiple outcomes examined, the results were interpreted with emphasis on effect sizes, 95% CIs, and consistency of findings rather than significance alone. As a supplementary exploratory analysis, between-group comparisons of VEQ subscale scores were performed using Wilcoxon rank-sum tests, with *P* values adjusted across the 3 VEQ subscales using the Benjamini-Hochberg FDR procedure.

For transparency, the repeated-measures ANOVAs and within-group pre-post analyses were repeated using absolute (nonnormalized) strength values (kgf) (Multimedia Appendix 1).

### Ethical Considerations

The Ethics Committee of Aichi Medical University approved the study protocol (approval number 2023‐586). All participants provided written informed consent before participation. This study was registered in the UMIN Clinical Trials Registry (UMIN000056456). Participant data were deidentified before analysis, and confidentiality was maintained throughout the study. Participants received a small honorarium in the form of a QUO CARD (a prepaid gift card widely used in Japan) valued at 1000 Japanese Yen (JPY; approximately US $6) for their participation. No identifiable images of individual participants are included in the manuscript or supplementary materials.

## Results

### Participant Flow

A total of 32 participants were included in the analysis (CLBP group: n=16; healthy controls: n=16). The participant flow diagram is shown in Figure 2. There were no missing data in the variables included in the present analyses.

**Figure 2.**
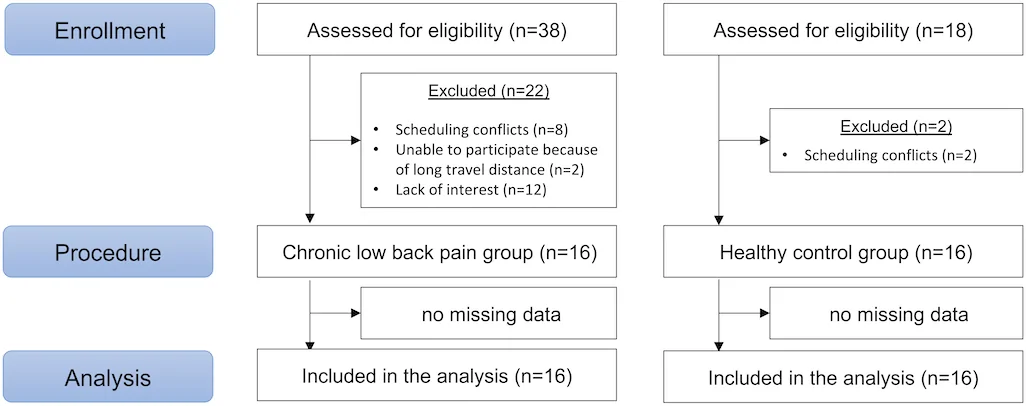
Participant flow diagram. Participants were assessed for eligibility and allocated to the chronic low back pain group or healthy control group. All 32 participants (16 per group) completed the study and were included in the analysis.

### Recruitment

Participant recruitment and data collection were conducted from November 2024 to March 2025.

### Statistics and Data Analysis

#### Participant Characteristics

The demographic and baseline characteristics are summarized in Table 1. Participants in the CLBP group exhibited long-standing pain with moderate intensity and mild disability, along with mild-to-moderate alterations in back-related body perception (Table 1). The median number of squats completed during the task was 8.5 (IQR 7‐10.25) in the CLBP group and 8 (IQR 7‐9.25) in the healthy control group. Thirty-one of the 32 participants reported noticing a change in the avatar’s muscularity, whereas one participant was uncertain whether a change had occurred.

#### Muscle Strength Outcomes

Using body-weight–normalized values, no significant group × time interactions were observed for handgrip, trunk extension, or knee extension strength (all *P*>.19) (Table 2 and Figure 3). Knee extension strength significantly increased from pre to post in both groups (main effect of time, *P*<.001), with moderate and comparable effect sizes in the CLBP group and healthy controls (Cohen *d*_av =0.43, 0.45).

**Table 2.** Immediate changes in body-weight–normalized muscle strength before and after the VR intervention. Values are presented as mean (SD) or mean difference (95% CI).

Variables and group	Pre-VR^a^ mean (SD)	Post-VR mean (SD)	Mean difference (95% CI)	Time (*P* value)	Group × time interaction (*P* value)	Post hoc *P* value (Bonferroni)	Cohen *d*_av
Handgrip strength (kgf/kg)							
CLBP^b^	0.51 (0.14)	0.53 (0.12)	0.01 (–0.01 to 0.04)	.04	.75	.54	0.11
HC^c^	0.49 (0.14)	0.51 (0.12)	0.02 (–0.001 to 0.04)	.04	.75	.12	0.15
Trunk extension strength (kgf/kg)							
CLBP	0.29 (0.06)	0.31 (0.05)	0.02 (0.001 to 0.05)	<.001	.19	.08	0.39
HC	0.28 (0.05)	0.32 (0.05)	0.04 (0.03 to 0.05)	<.001	.19	<.001	0.77
Knee extension strength (kgf/kg)							
CLBP	0.57 (0.15)	0.63 (0.15)	0.06 (0.02 to 0.11)	<.001	.28	.02	0.43
HC	0.63 (0.20)	0.72 (0.20)	0.09 (0.06 to 0.12)	<.001	.28	<.001	0.45

a VR: virtual reality.

b CLBP: chronic low back pain.

c HC: healthy control.

**Figure 3.**
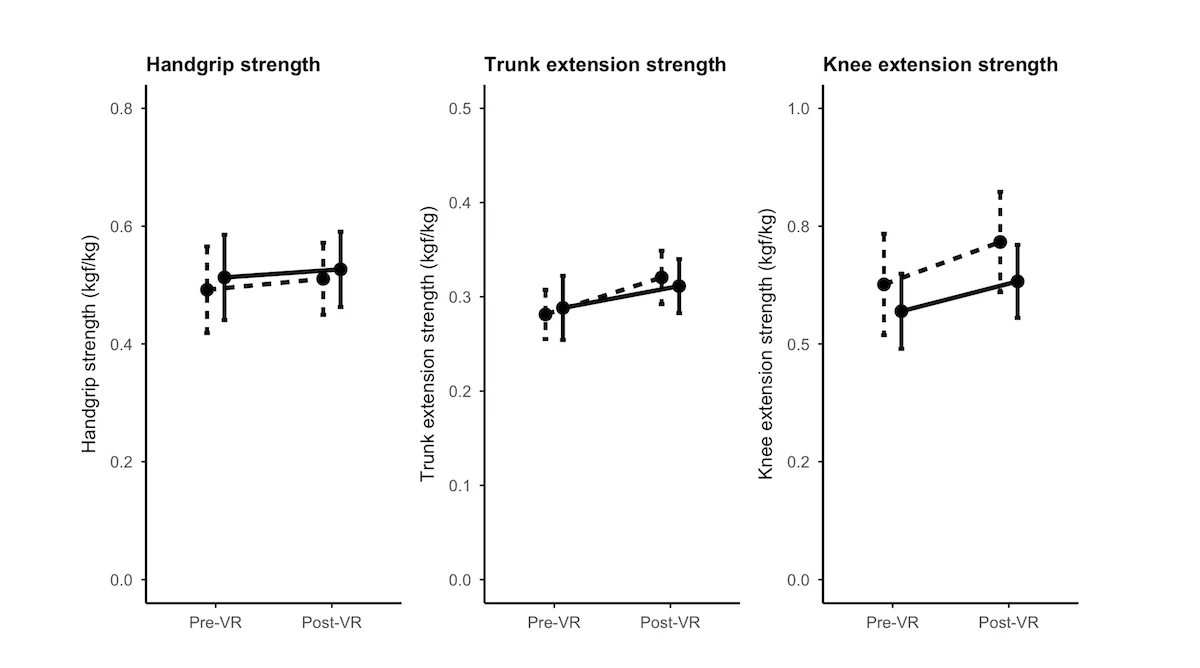
Changes in muscle strength before and after the virtual reality–based squat task. Mean values with 95% CIs are shown for handgrip, trunk extension, and knee extension strength in the chronic low back pain group and healthy controls. Strength was assessed immediately before and after the virtual reality session. All strength values were normalized to body weight (kgf/kg). Solid lines indicate the chronic low back pain group and dashed lines indicate healthy controls. VR: virtual reality.

Values are presented as mean (SD) or mean difference (95% CI). Strength values were normalized to body weight (kgf/kg). Mean differences indicate post-VR minus pre-VR values. Time effects and group × time interactions were evaluated using 2-way repeated-measures analysis of variance with type III sums of squares. The 95% CIs for mean differences are unadjusted; post hoc within-group *P* values were Bonferroni adjusted. Within-group effect sizes are expressed as Cohen *d*_av.

The trunk extension strength also showed a significant main effect of time (*P*<.001). Post hoc pairwise comparisons indicated a significant pre-post increase in healthy controls (Cohen *d*_av =0.77; *P*<.001), whereas the CLBP group showed a nonsignificant pre-post increase (Cohen *d*_av =0.39; *P*=.08).

Handgrip strength showed a significant main effect of time (*P*=.04); however, post hoc comparisons did not reveal significant pre-post changes within either group (CLBP: *P*=.54; healthy controls: *P*=.12), and the corresponding within-group effect sizes were small (Cohen *d*_av =0.11‐0.15). For transparency, the results based on absolute (nonnormalized) strength values are provided in Table S1 in Multimedia Appendix 1.

Associations between muscle strength changes and virtual embodiment (Table 3).

**Table 3.** Correlations between changes in muscle strength and Virtual Embodiment Questionnaire subscales. Δ denotes the within-session change (post–virtual reality − pre–virtual reality).

	CLBP^a^			HC^b^		
Δ Strength and VEQ^c^	ρ^d^ (95% CI)	*P* value	FDR^e^-adjusted *P* value	ρ (95% CI)	*P* value	FDR-adjusted *P* value
Δ Handgrip strength (kgf)						
Ownership	0.35 (–0.26 to 0.84)	.18	.32	0.30 (–0.27 to 0.75)	.26	.91
Agency	0.14 (–0.46 to 0.66)	.66	.69	0.05 (–0.48 to 0.55)	.87	.91
Change	0.04 (–0.48 to 0.51)	.88	.88	0.23 (–0.40 to 0.83)	.38	.91
Δ Trunk extension strength (kgf)						
Ownership	0.50 (0.02 to 0.82)	.05	.15	0.06 (–0.49 to 0.64)	.82	.91
Agency	0.55 (0.06 to 0.84)	.03	.12	–0.06 (–0.62 to 0.53)	.84	.91
Change	0.76 (0.41 to 0.94)	.001	.006	0.27 (–0.39 to 0.76)	.32	.91
Δ Knee extension strength (kgf)						
Ownership	0.28 (–0.29 to 0.75)	.29	.43	0.10 (–0.53 to 0.68)	.70	.91
Agency	0.18 (–0.41 to 0.67)	.50	.64	–0.03 (–0.61 to 0.55)	.91	.91
Change	−0.36 (–0.76 to 0.17)	.17	.32	0.10 (–0.49 to 0.66)	.71	.91

a CLBP: chronic low back pain.

b HC: healthy control.

c VEQ: Virtual Embodiment Questionnaire.

d *ρ*: Spearman rank correlation coefficient.

e FDR: false discovery rate.

In the CLBP group, a significant positive correlation was observed between the change in trunk extension strength and the VEQ Change score (ρ=0.76, 95% CI 0.41-0.94, FDR-adjusted *P*=.006; *P*=.001). Correlations with VEQ Ownership (ρ=0.50, 95% CI 0.02-0.82, FDR-adjusted *P*=.15; *P*=.05) and VEQ Agency (ρ=0.55, 95% CI 0.06-0.84, FDR-adjusted *P* =.12; *P*=.03) showed similar positive trends, although these did not remain significant after FDR correction (Figure 4). No significant correlations were found in the healthy controls. To provide additional context for the embodiment-related findings, we also performed exploratory between-group comparisons of VEQ subscale scores. No significant between-group differences were observed after FDR correction (Table S2 in Multimedia Appendix 1).

**Figure 4.**
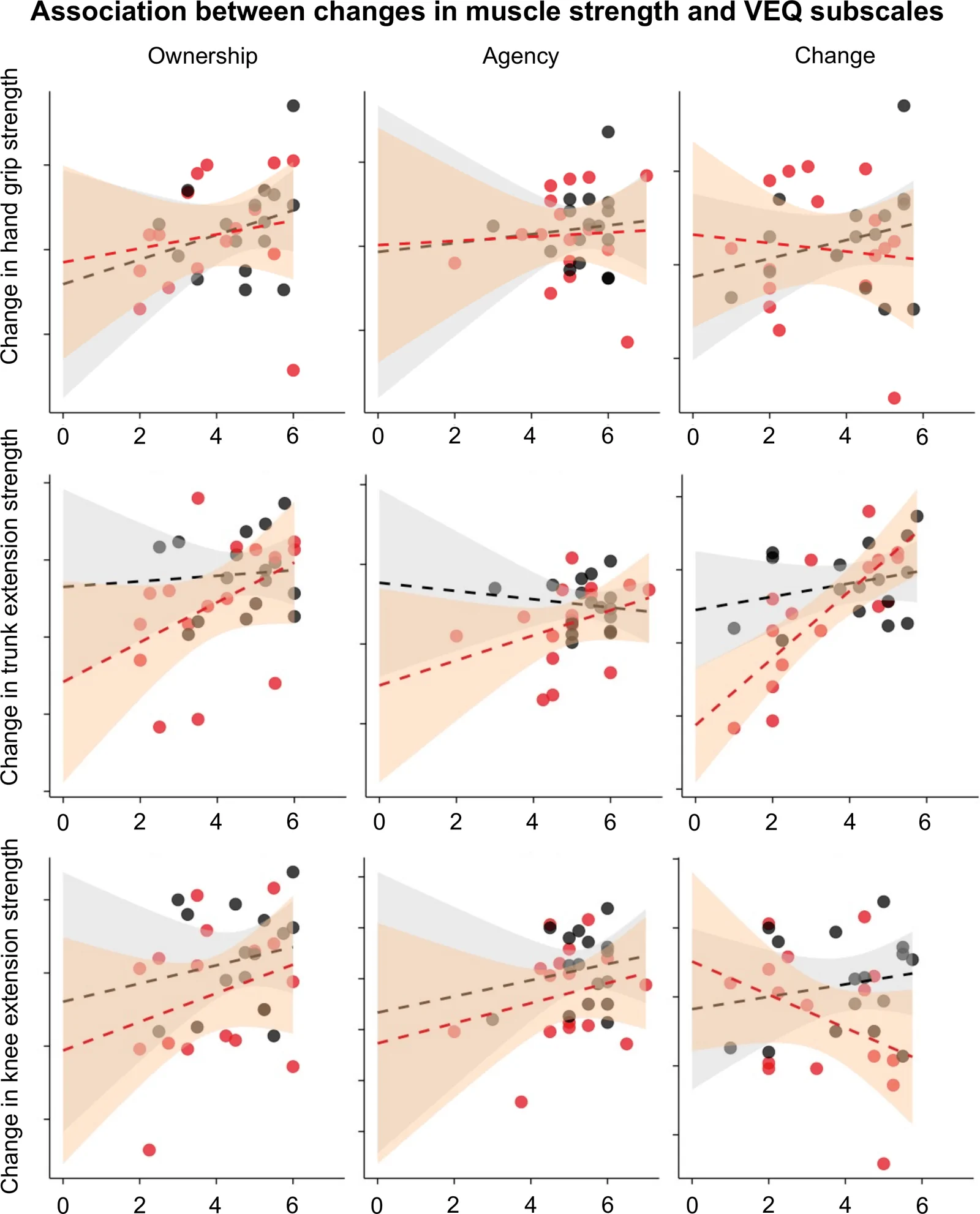
Associations between changes in muscle strength and virtual embodiment. Scatterplots depict the associations between changes in muscle strength and scores on the Virtual Embodiment Questionnaire subscales. Red points indicate the chronic low back pain group, and black points indicate healthy controls. Spearman rank correlation coefficient was used. Dashed lines indicate fitted regression trends, and shaded areas represent 95% CIs. VEQ: Virtual Embodiment Questionnaire.

#### Pain and Fear Ratings in the CLBP Group

Pain intensity showed a significant change over time (*χ*²_2_=11.48, Kendall *W*=0.359; *P*=.003). Pain decreased during the VR-based squat task compared with the pre-VR levels (*P*=.02), whereas pain intensity after the task did not significantly differ from either the pre-VR or during-VR levels (all *P*≥.12) (Figure 5A).

Fear of squat motion also demonstrated a significant overall change over time (*χ*²_2_=7.92, Kendall *W*=0.248; *P*=.02); however, no specific pairwise comparison reached statistical significance after correction (all *P*≥.10) (Figure 5B).

**Figure 5.**
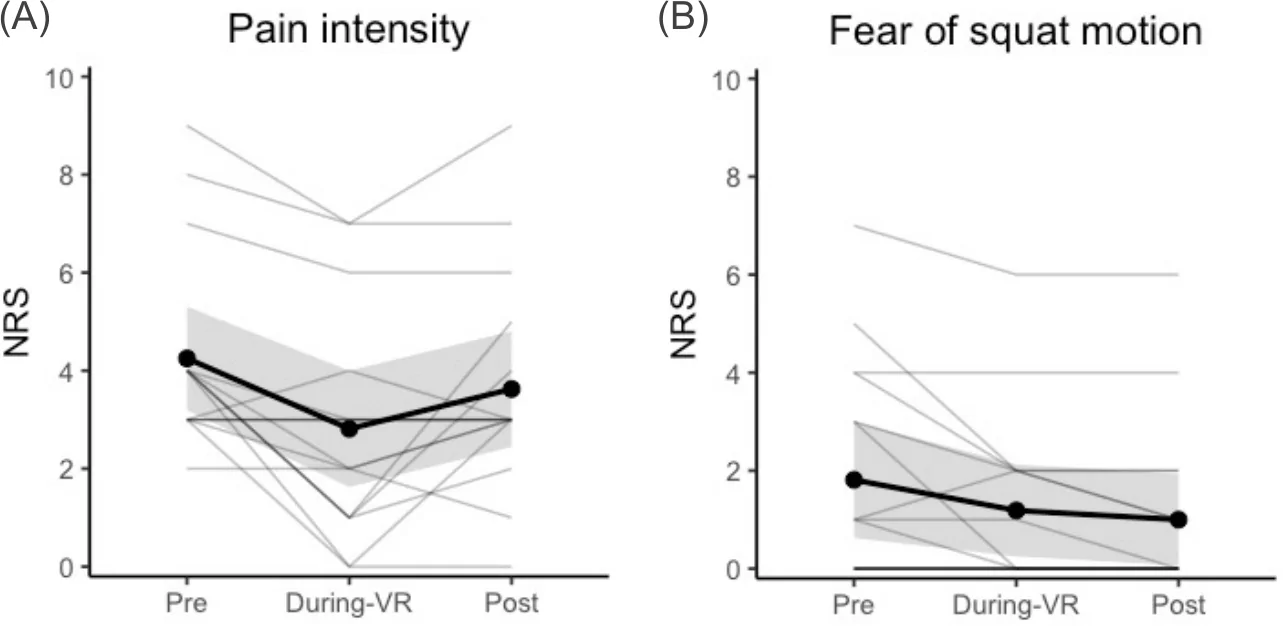
Changes in pain intensity and fear ratings during the virtual reality–based squat task in the chronic low back pain group. Thin lines represent individual participant trajectories (spaghetti plot). Bold lines indicate chronic low back pain group mean values, and shaded areas denote 95% CIs. Pain intensity and fear ratings were assessed at pre-virtual reality, during-virtual reality, and post-virtual reality time points. NRS: Numerical Rating Scale; VR: virtual reality.

## Discussion

### Support of Original Hypotheses

Overall, the findings partially supported our hypotheses. Task-relevant trunk and knee extension strength showed immediate time-related increases after the VR-based squat task, whereas handgrip strength showed only a small overall time-related change. Pain intensity did not worsen, and it decreased during the VR session in the CLBP group. In addition, the association between trunk strength change and VEQ change in the CLBP group suggested a possible embodiment-related within-group association. However, because no significant group × time interactions were observed and no neutral-avatar or no-avatar control condition was included, these findings should be interpreted as exploratory and hypothesis-generating rather than as evidence of a causal or CLBP-specific effect.

### Similarity of Results

The pattern of muscle strength changes observed in this study may provide preliminary insight into how a muscular-avatar exercise task relates to objective muscle performance. Trunk and knee extension strength showed time-related increases, whereas the overall change in handgrip strength was smaller. Because trunk and knee extension were more directly involved in the squat task than handgrip strength, this pattern may be compatible with a greater change in task-relevant motor output. However, the magnitude of change was not directly compared across muscle groups, and the absence of a neutral-avatar or no-avatar control condition means that the observed changes cannot be separated from repeated muscle activation, familiarization, warm-up, generic visual feedback, or attentional engagement.

Previous studies in healthy participants suggest that the effects of muscular avatars on exercise-related responses are heterogeneous rather than uniformly performance-enhancing. Kocur et al [31] reported that embodying a muscular avatar reduced perceived exertion during an isometric task and was associated with greater grip strength in male participants, although the performance effects were not consistent across sex or avatar conditions. Lin et al [32] subsequently found that embodying a six-pack avatar was associated with lower physical activity, no significant change in perceived exertion, and sex-dependent effects on exercise self-efficacy and self-concept. More recently, Kocur et al [33] reported that heart rate increased more slowly during rowing with a muscular than with a nonmuscular avatar, whereas perceived exertion did not differ significantly between conditions. Taken together, these findings suggest that avatar muscularity may influence perceptual, behavioral, or physiological responses during exercise, but that its effects depend on the task, outcome, and participant characteristics. These observations are broadly compatible with the Proteus effect, whereby avatar appearance may shape users’ self-perception and behavior [14,15], but they do not indicate a uniform or context-independent enhancement of physical performance.

Research applying virtual embodiment to CLBP has primarily examined pain-related, body-perception-related, and psychological outcomes rather than objective muscle performance. In a two-patient case series, Trujillo et al [34] reported short-term reductions in pain intensity and pain catastrophizing following a multicomponent program combining virtual embodiment, graded motor imagery, and functional exercise. Harvie et al [17] subsequently found that embodying superhero-like avatars produced immediate improvements in body image, including perceived strength and confidence, but did not significantly change pain, fear of movement, or handgrip strength after a single session. Nishigami et al [35] further showed that a strong, fit-back illusion was associated with lower pain and fear and greater perceived back strength and confidence in a participant who successfully embodied the illusion, whereas little change occurred in a participant who did not embody it. Taken together, these studies suggest that virtual embodiment can modify pain-related and capability-related self-perceptions in CLBP, whereas its effects on objective muscle performance remain insufficiently understood. By examining objective muscle strength together with subjective embodiment, the present study extends previous work that has primarily focused on subjective pain-related and body-perception-related outcomes.

### Interpretation

#### Embodiment-Related Associations in CLBP

Against this background, the observed association between trunk strength change and VEQ change warrants consideration within the motor-control and body-perception characteristics of CLBP. In individuals with CLBP, trunk motor output may be influenced by pain-related motor adaptation, heterogeneous motor control strategies, and altered lumbopelvic movement patterns [7,8,36]. In this study, participants with CLBP who reported stronger virtual embodiment, particularly greater momentary changes in perceived bodily capability, tended to show larger increases in trunk extension strength. However, given the absence of a significant group × time interaction, the modest sample size, and the multiple correlation analyses performed, this finding should be interpreted as an exploratory within-group association rather than as evidence of a differential, CLBP-specific, or causal effect. This interpretation is consistent with previous reports showing that motor control strategies in CLBP are heterogeneous, ranging from excessive co-contraction to hypoactivation, with distinct functional consequences [8].

This association may also be considered within the broader framework of virtual embodiment, which has been conceptualized as a multidimensional experience involving body ownership, agency, and self-location [37]. Immersive VR and virtual embodiment may influence pain-related and movement-related responses by temporarily altering body representation and bodily self-perception through multisensory integration [38]. Embodiment and body transformation illusions have also been proposed as potentially relevant approaches for targeting altered bodily perceptions in chronic pain [39]. In CLBP, disturbances in body perception, including impaired tactile acuity and distorted back-specific body image, have been reported [12,27,28,40]. Altered sensorimotor integration may also be relevant to the relationship between body perception and motor output in CLBP [41]. Recent studies further suggest that virtual visual-proprioceptive manipulation can alter movement-evoked pain perception and that VR-based interventions, including programs combining movement exercises and body illusions and other novel VR approaches, may be feasible in chronic back pain and CLBP [18,19,42]. In this context, the association between trunk strength change and VEQ change may reflect a relationship between virtual embodiment, task-specific bodily self-perception, and motor output. This provides a plausible hypothesis that visually guided support of body perception may help facilitate voluntary motor output during exercise in some individuals with CLBP. However, because no neurophysiological measures were obtained, the mechanisms underlying this association remain speculative and cannot be directly determined from the present study. Individual differences in embodiment susceptibility may therefore represent one factor influencing responsiveness to visually augmented exercise-based rehabilitation and warrant further investigation in future studies.

### Pain Reduction and Attentional Shift

In the CLBP group, pain intensity decreased during VR exposure. This transient, within-session reduction in pain is consistent with previous evidence that VR distraction can reduce pain during and immediately after exercise in individuals with CLBP, as well as broader evidence supporting short-term analgesic effects of VR [43,44]. A replicated single-case experimental study reported short-term improvements in pain-related outcomes using a novel VR intervention for people with CLBP [42], whereas a recent systematic review and meta-analysis suggested that VR-based training may improve pain-related outcomes in CLBP, although the durability and mechanisms of these effects remain insufficiently established [45,46]. Given evidence of attentional bias toward pain-related information in individuals with chronic pain [47,48], interventions that redirect attention away from pain-related cues may be clinically relevant. In this context, VR may serve as a brief attentional primer to facilitate engagement in feared movements such as squatting, functioning as a transient adjunct to rehabilitation rather than as a stand-alone analgesic. Taken together, these findings suggest that attentional distraction may represent one pathway through which VR influences pain, while transient changes in body representation and perceived physical capability may represent additional mechanisms worthy of further investigation.

### Generalizability

This proof-of-concept study has several limitations that should be considered when interpreting the generalizability of the findings. First, we lacked a neutral or fixed-avatar or no-avatar control condition; therefore, the observed effects cannot be attributed specifically to the manipulation of avatar muscularity and cannot be separated from practice effects, generic visual feedback, or attentional factors. Although 31 of 32 participants reported noticing a change in the avatar’s muscularity, no objective or quantitative manipulation check was conducted to determine whether the intended psychological manipulation was successful. Second, this was a single-session proof-of-concept study with a modest sample size; therefore, the durability, reproducibility, and statistical precision of the findings are limited. Although FDR correction was applied where appropriate, the number of comparisons increases the risk of type I error [49]. In addition, the modest sample size limited statistical power and increased the risk of type II error [50]. Therefore, nonsignificant findings should be interpreted with caution.

Third, squat depth, cadence, fatigue, and motivation were not objectively controlled or logged. Although the specific study hypothesis was not disclosed, participants were aware that they were taking part in a VR-based exercise study, which may have influenced self-reported outcomes through expectancy or response bias [51]. Repeated performance of the task and strength assessments may also have introduced practice or familiarization effects [52]. In addition, recruitment from a single center may limit the generalizability of the findings to other populations and clinical settings [53]. Fourth, the Japanese version of the VEQ provided by the questionnaire developers was used [29,30]. However, to our knowledge, a formal psychometric validation of the Japanese version has not been published; therefore, measurement error cannot be excluded.

Fifth, handgrip strength was assessed only on the right side, and hand dominance was not formally evaluated; therefore, potential effects of hand dominance cannot be excluded [54]. We did not collect electromyography or other physiological or behavioral markers to triangulate the neuromuscular mechanisms. Finally, the avatar was driven by a markerless tracking system, and end-to-end latency, joint-position accuracy, and occlusion were not quantified. Any mismatch between real and rendered movements, including temporal or spatial discrepancies due to tracking limitations, may reduce visuomotor congruence and thereby weaken the sense of agency and, potentially, body ownership during virtual embodiment [55].

### Implications for Future Research and Clinical Practice

From a clinical perspective, approaches that combine physical exercise with visually augmented feedback designed to engage body representation and perceived physical capability warrant further investigation in CLBP [17,56]. The present findings do not establish the clinical efficacy of this approach. Rather, they support the hypothesis-generating value of visually augmented exercise strategies that may help some individuals engage more effectively in movement when motor output is constrained by pain, fear, or altered body perception.

Future studies should move beyond proof-of-concept designs by directly testing the mechanisms that may underlie avatar-related changes in body representation and motor output. Specifically, studies with neutral-avatar or no-avatar control conditions are needed to distinguish avatar-specific effects from practice, attentional, or general visual-feedback effects. In addition, objective neurophysiological and psychophysical measures may help clarify the mechanisms underlying VR-related changes in pain and behavior [57]. Such approaches may include electromyography, electroencephalography, functional near-infrared spectroscopy, or autonomic indices to examine potential changes in motor activation, neural processing, or nociceptive processing. Future research should also examine whether individual variability in embodiment-related responses is associated with responsiveness to visually augmented exercise-based rehabilitation.

### Conclusions

This exploratory proof-of-concept, single-session study observed immediate time-related increases in task-relevant muscle strength after a VR-based squat task involving embodiment of a progressively muscular avatar. However, because these changes occurred in both groups and no significant group × time interactions were found, the findings should not be interpreted as evidence of a causal or CLBP-specific effect. By examining objective muscle strength outcomes alongside subjective embodiment, this study extends previous VR research in chronic pain that has primarily focused on pain intensity, fear, or body perception. The association between trunk strength change and VEQ change in the CLBP group provides hypothesis-generating evidence that visually guided modulation of body perception may be relevant to individual variability in motor output during exercise. These preliminary findings suggest that visual approaches targeting body perception may warrant further investigation as potential adjuncts to exercise-based rehabilitation for individuals whose movement engagement is constrained by pain, fear, or altered body perception.

## Supplementary material

10.2196/90389 Multimedia Appendix 1 Absolute muscle strength changes and Virtual Embodiment Questionnaire subscale comparisons.

## Abbreviations

CLBP: chronic low back pain
FDR: false discovery rate
FreBAQ: Fremantle Back Awareness Questionnaire
NRS: Numerical Rating Scale
RDQ: Roland-Morris Disability Questionnaire
VEQ: Virtual Embodiment Questionnaire
VR: virtual reality
