# Supplementary material for: Association Between Embodiment of a Muscular Avatar and Muscle Performance in Virtual Reality for Chronic Low Back Pain: Exploratory Pre-Post Study
Source: JMIR Serious Games. 2026 Oct 9;14:e90389. doi: 10.2196/90389 (PMC13652391; doi:10.2196/90389)
Supplement: Supplementary file 1 [file games-v14-e90389-s001.docx]

Table S1. Immediate changes in absolute muscle strength between pre-VR and post-VR

| Variables | Group | Pre-VR,  mean (SD) | Post-VR,  mean (SD) | Mean difference  (95% CI) | Time effect  (*P* value) | Time effect  (partial *η²*) | Group effect  (*P* value) | Interaction  (*P* value) | Post hoc *P*  (Bonferroni) | Cohen *d*_av |
| --- | --- | --- | --- | --- | --- | --- | --- | --- | --- | --- |
| Handgrip strength  (kgf) | CLBP | 30.81 (10.90) | 31.49 (9.94) | 0.68 (-0.79 to 2.15) | .06 | .12 | .66 | .66 | .34 | 0.06 |
|  | Healthy controls | 32.38 (11.82) | 33.46 (10.53) | 1.08 (-0.11 to 2.26) |  |  |  |  | .07 | 0.09 |
| Trunk extension strength (kgf) | CLBP | 17.04 (4.34) | 18.31 (3.84) | 1.27 (-0.10 to 2.64) | <.001 | .45 | .24 | .12 | .13 | 0.30 |
|  | Healthy controls | 18.43 (4.94) | 20.88 (5.12) | 2.46 (1.65 to 3.26) |  |  |  |  | <.001 | 0.47 |
| Knee extension strength (kgf) | CLBP | 34.74 (13.69) | 38.24 (13.56) | 3.51 (0.95 to 6.07) | <.001 | .57 | .18 | .12 | .02 | 0.25 |
|  | Healthy controls | 41.65 (18.05) | 47.58 (18.56) | 5.93 (4.03 to 7.83) |  |  |  |  | <.001 | 0.31 |

Values are presented as mean (SD) or mean difference (95% CI). Two-way repeated-measures ANOVA (group × time, type III) evaluated the time, group, and group × time interaction effects. Effect sizes for the time effect are reported as partial η². Within-group effect sizes are reported as Cohen dav (standardized mean change using the average of pre- and post-VR SDs). Post hoc within-group P values are Bonferroni-adjusted (2-tailed *α*=.05). Muscle strength is expressed in kgf and is not normalized to body weight.

Abbreviations: CLBP, chronic low back pain; VR, virtual reality.

Table S2. Exploratory between-group comparisons of VEQ subscale scores

| VEQ subscale | Healthy controls, mean (SD) | CLBP, mean (SD) | *P* value | FDR-adjusted *P* value |
| --- | --- | --- | --- | --- |
| Ownership | 4.66 (1.08) | 3.89 (1.41) | .14 | .21 |
| Agency | 5.36 (0.79) | 4.92 (1.14) | .09 | .21 |
| Change | 4.05 (1.48) | 3.38 (1.45) | .22 | .22 |

Values are presented as mean (SD). Between-group comparisons were performed using Wilcoxon rank-sum tests. *P* values were adjusted using the Benjamini-Hochberg false discovery rate procedure across the three VEQ subscales.

Abbreviations: CLBP, chronic low back pain; FDR, false discovery rate; VEQ, Virtual Embodiment Questionnaire.
